# Structural, Dynamical, and Energetical Consequences of Rett Syndrome Mutation R133C in MeCP2

**DOI:** 10.1155/2015/746157

**Published:** 2015-04-05

**Authors:** Tugba G. Kucukkal, Emil Alexov

**Affiliations:** Computational Biophysics and Bioinformatics, Department of Physics, Clemson University, Clemson, SC 29634, USA

## Abstract

Rett Syndrome (RTT) is a progressive neurodevelopmental disease affecting females. RTT is caused by mutations in the* MECP2* gene and various amino acid substitutions have been identified clinically in different domains of the multifunctional MeCP2 protein encoded by this gene. The R133C variant in the methylated-CpG-binding domain (MBD) of MeCP2 is the second most common disease-causing mutation in the MBD. Comparative molecular dynamics simulations of R133C mutant and wild-type MBD have been performed to understand the impact of the mutation on structure, dynamics, and interactions of the protein and subsequently understand the disease mechanism. Two salt bridges within the protein and two critical hydrogen bonds between the protein and DNA are lost upon the R133C mutation. The mutation was found to weaken the interaction with DNA and also cause loss of helicity within the 141-144 region. The structural, dynamical, and energetical consequences of R133C mutation were investigated in detail at the atomic resolution. Several important implications of this have been shown regarding protein stability and hydration dynamics as well as its interaction with DNA. The results are in agreement with previous experimental studies and further provide atomic level understanding of the molecular origin of RTT associated with R133C variant.

## 1. Introduction

Rett Syndrome (RTT) is an X-linked severe neurodevelopmental disorder [1–5]. It is a progressive disease after onset and especially affects the expressive language and hand use [6–10]. RTT affects 1 in 10,000 females with 20,000 RTT patients in the US and 50,000 worldwide [7]. The mutations in MeCP2 are the major cause of RTT as they have been detected in more than 90% of classical RTT patients [8, 11]. In addition, the MeCP2 mutations are associated with X-linked mental retardation and other neurological disorders [8, 12]. MeCP2 is a member of the methyl-CpG-binding domain (MBD) family of proteins and has three major domains: the abovementioned MBD, the transcriptional repression domain (TRD), and the C-terminal domain (CTD). MeCP2 binds to symmetrical methylated 5′ CpG pairs through its MBD with a preference for A/T-rich motifs [13]. It also belongs to intrinsically disordered family of proteins [14] and therefore binds to a number of other partners through its disordered regions, which span about 65% of the protein [15]. In general, MeCP2 serves diverse functions in gene regulation and chromatin organization and particularly it is a transcriptional repressor that mediates gene silencing through binding to methylated DNA [11]. However, recent studies indicate that it also can act as an activator [11]. In the body, it is distributed to all tissues but particularly abundant in brain [11].

There have been a number of RTT-causing mutations identified at different regions of the 486-residue MeCP2 protein. Considering the mutations with a frequency of more than 0.05%, the 20% of the RTT cases are caused by mutations in the MBD domain of MeCP2. Also, the deleterious mutations are responsible for 27% of the cases and the missense mutations in parts of MeCP2 other than the MBD domain are responsible for 14% of RTT cases [16].

Here we focus on the mutations occurring in MBD and particularly studied here is the R133C mutation [17, 18]. The Arg 133 is one of the two residues that make direct contacts with DNA and R133C mutation is the second most common mutation in MBD affecting more than 4% of all RTT cases [16]. Extensive molecular dynamics (MD) studies have been performed on both wild-type (WT) and R133C mutant to understand the effects of mutation on protein structural, dynamical, and energetical properties at the molecular level. The comparative MD studies reveal important details on how R133C impacts MBD and MBD-DNA recognition. The calculated effects are consistent with previously published experimental data while providing further atomic level details of the molecular origin of disease associated with the R133C variant.

## 2. Methods

### 2.1. Structure Preparation

The X-ray structure of MBD of MeCP2 bound to DNA, PDB ID 3C2I [19], was used as the initial structure. This structure has the mutation A140M; therefore, first the Met at position 140 was mutated back to Ala ensuring the canonical sequence for the MBD. Then, the R133C mutation was introduced on this structure. The WT and R133C structures were then independently subjected to further preparation. First, the missing hydrogen atoms were added and then the structures were solvated with TIP3 water molecules [20] together with sodium and chloride ions to ensure 0.15 M concentration and neutral systems. The ions were placed using the Autoionize Plugin in the VMD software [21]. A periodic box with edges extending at least 10 Å from solute atoms was used, thus ensuring 3-4 hydration layers in each direction. The initial periodic cell size was about 80 × 53 × 78 Å^3^ with ~31,000 atoms in each system. All mutations and other structural preparations were performed utilizing the VMD program [21].

### 2.2. Molecular Dynamics

Langevin dynamics with periodic boundary conditions was performed in the NPT (constant pressure, constant temperature) ensemble using the CHARMM22 force field for proteins [22] with CMAP corrections [23, 24] and the CHARMM27 force field [25, 26] for the DNA. vdW interactions were truncated with a switching function in 10 Å distance with 8 to 10 Å cutoff. Electrostatic interactions were truncated with particle mesh Ewald (PME) [27] and 1 Å grid spacing was used. A temperature of 298 K was maintained using Langevin dynamics, with a damping constant of 1 ps^−1^. 1 atm pressure was maintained using the Nosé-Andersen Langevin piston [28, 29]. First, the systems were relaxed for 4000 steps and then MD simulations were run with a time step of 1 fs for ~5 ns. Then, a time step of 1.5 fs was used and the simulations were run for 220 ns in total. The lengths of all bonds involving hydrogens were constrained with the RATTLE algorithm [30] as implemented in NAMD [31]. All simulations were performed using NAMD.

### 2.3. Analysis of MD Energies and Trajectories

All the analyses were performed utilizing VMD unless otherwise stated. Root mean square fluctuations (RMSF) of *C*_*α*_ atoms were calculated using the trajectory after the 30th ns (last 190 ns) ensuring that the systems were equilibrated. The solvent accessible surface area (SASA) was calculated with a 1.4 Å probe radius. Contact area was calculated as

(1) $$\begin{array}{c} \text{Contact}\;\text{area}=\;\frac{\text{SAS}{\text{A}}_{\text{protein}}+\text{SAS}{\text{A}}_{\text{DNA}}-\text{SAS}{\text{A}}_{\text{complex}}}{2}. \end{array}$$

Hydrogen bonding criteria used in analyses are the donor-acceptor distance of 3 Å or less and the donor-hydrogen-acceptor angle of 20 degrees or less. Also, a 3.2 Å cutoff of oxygen-nitrogen is used for salt bridges. The time-dependent distance profile of residue 133 and mC33 was calculated by taking the distance between center of mass of residue 133 of the protein and the center of mass mC33 of the DNA. All the analyses described so far have been performed using the structures saved every 3 ps.

Interaction energy (van der Waals and electrostatic) was calculated for structures saved every 200 ps with the same cutoff as the MD simulations. Cluster analysis was performed with a 2 Å cutoff of RMSD for the residues 99 to 158. Secondary structure analysis was based on the DSSP algorithm [32] as implemented in the WHATIF program [33].

## 3. Results and Discussion

**Figure 1:**
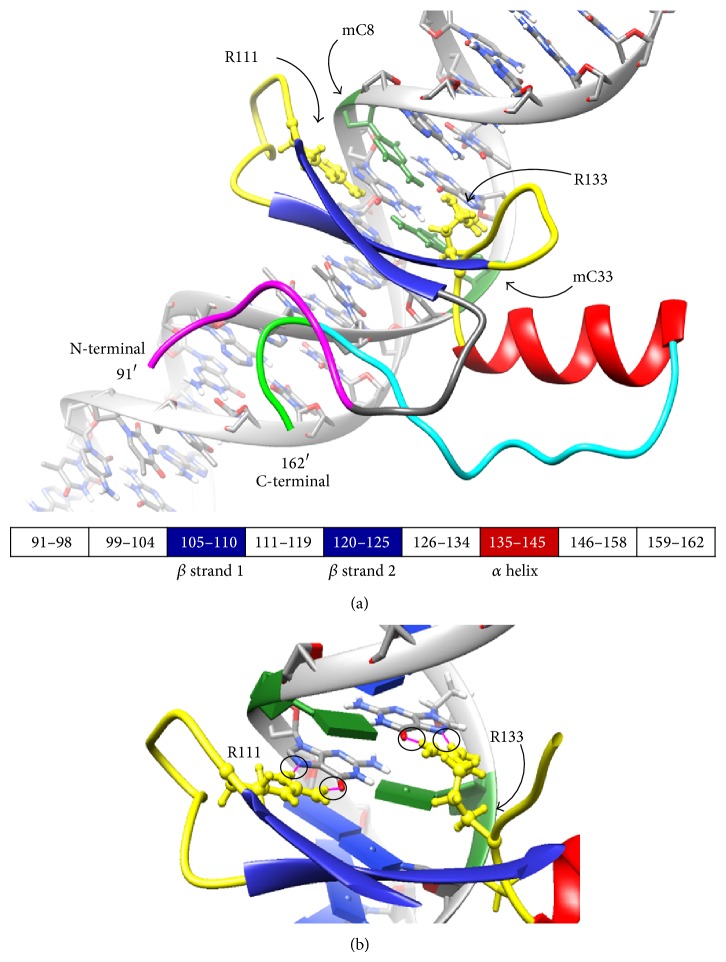
Initial structure of WT protein with DNA (PDB ID: 3C2I). The two Arg residues (R133 and R111) are shown in yellow. The two methylated Cyt residues of DNA are shown in green. The 9 regions of protein are colored differently and sequence breakdown with structured regions is shown at the middle panel. The hydrogen bonding between R133 and R111 and DNA is shown (magenta colored lines in circles) at the bottom image.

The crystal structure of MBD of MeCP2 bound to DNA revealed important structural information regarding its binding to DNA as well as its fold. The protein has two major *β* strands and an *α* helix forming a wedge-like shape with two major coil (turn) regions that make direct contacts with the two symmetrical methylated cytosine (mC) of methylated-CpG DNA. As shown in Figure 1(a), the crystal structure indicates that the two Arg residues, Arg133 and Arg111, form direct hydrogen bonds with DNA. The frequent RTT mutation R133C has immediate structural consequences such as the loss of the direct hydrogen bond with DNA as shown in Figure 1(b). 220 ns long MD simulations of WT and R133C systems reveal important consequences of the mutation in structure, dynamics, and interactions of MBD of MeCP2.

The rest of the section is organized into three major components: (a) structural and dynamical analysis of the systems, (b) solvent accessible surface area (SASA) and radius of gyration (*R*_gyr_) analysis, and (c) analysis of the specific interactions of protein, DNA, and water through hydrogen bonding and energetics calculations.

**Figure 2:**
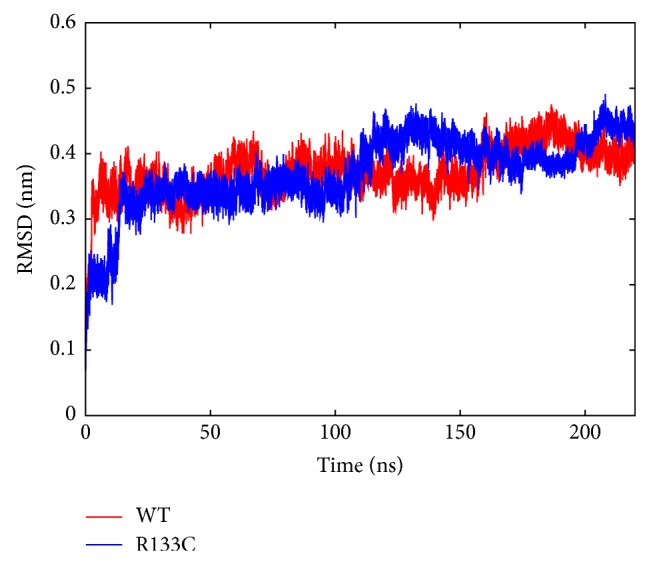
Time-dependent RMSD profile of residues 99–158 for WT (red) and R133C (blue).

**Figure 3:**
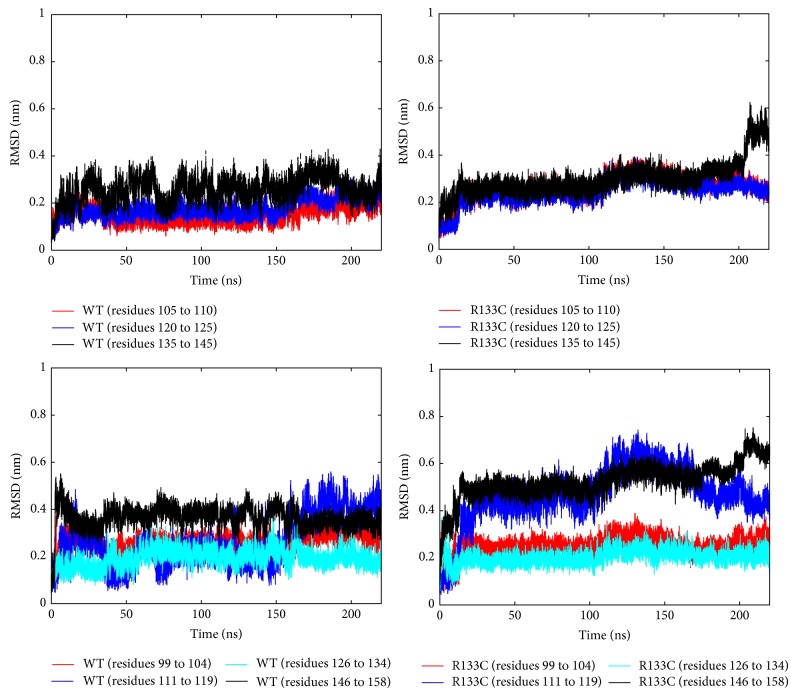
Time-dependent RMSD changes of loop regions (bottom panels) and structured regions (top panels) for WT (left panels) and R133C (right panels).

*(a) Impact of R133C Mutation on Structure and Conformational Dynamics*. First, time-dependent root mean square deviations (RMSDs) of the MBD of MeCP2 (WT and R133C) from their initial structures were computed. Monitoring the RMSD evolution of the protein can give insights into its structural conformation and also the structural integrity of biomolecules in the simulation system. Based on the change in RMSD, the protein (MBD of MeCP2) sequence was divided into 9 regions. Not surprisingly, the coiled regions at the beginning and end of the sequence (91–98 and 159–162) were found to be excessively floppy; therefore the RMSD of the whole protein, Figure 2, was obtained by leaving these regions out. The RMSD change flattens after about 20 ns and the variance stays under 0.1 nm until around 110 and 160 ns for WT and R133C, after which larger structural changes seem to take place. To pinpoint the source of these changes, the RMSD change over time for structured and coiled regions as shown in Figure 3 was examined. For the WT, the source of the changes was found to take place mostly in the coiled regions and visual inspection of MD trajectories reveals flipping of coiled regions. Although the R133C structure shows the same trend in general, it also has a drastic RMSD change in the helical region after ~200 ns.

The structures were examined more closely by performing cluster analysis (based on RMSD) for the WT and R133C MD trajectories for the sequence regions between 99 and 158. In each case, the structures were grouped in five (colored as yellow, purple, orange, green, and turquoise as shown in the first column of Figure 8) and representative structures were taken from each group. As the RMSD suggests in both cases, the structure remains the same for a long period of time (~20–100 ns); therefore one of the groups in the cluster has a large number of structures (green), for which 4 representative structures were considered. Then, the representative structures were subjected to a secondary structure analysis based on the DSSP algorithm. These secondary structure analysis results are shown in Figure 8. Taken together with the time-dependent RMSD profiles, the origin of the possible structural change for the WT is the loss of a very short strand region (residues 131 and 132). However, the abrupt change in the RMSD profile of the helical region of R133C as shown in Figure 3 top right panel originates from a significant loss of helicity in the 142–144 region (Figure 8). Based on this analysis, unlike the WT, the mutant was found to have preserved the short strand region (residues 131 and 132). Having discussed the structural analysis, we note that the results are being reported from a single trajectory.

The circular dichroism (CD) data indicated previously that the methylated DNA-induced stabilization for MBD of MeCP2 was less in the R133C mutant compared to WT [15]. In other words, the percent helicity was shown to increase ~5% in WT upon binding to methylated DNA (mDNA); however, the R133C mutant MBD did not show this structural enhancement upon binding to mDNA. Also, experiments suggested that the R133C mutation caused the loss of specificity for mDNA but did not abolish binding altogether [15, 34] as seen in the current MD results. Therefore, our results suggesting loss of helicity in the presence of methylated DNA and maintenance of binding upon mutation are consistent with these previous findings about the secondary structure of MBD upon mutation.

It is quite possible that the loss of helicity or reduced mDNA-induced stabilization may originate from altered DNA-MBD interactions particularly at the mutation site 133. To verify this, the DNA-protein interactions were analyzed in detail in the last section.

Before moving on to altered interactions and SASA changes, another structural property of the protein, the salt bridges were monitored particularly in the vicinity within 6 Å of R133 and within 6 Å of R111. One salt bridge in each region was lost upon R133C mutation as seen in Table 1. We speculate that the loss of the salt bridge between E137 and R133 might contribute to the loss of helicity in 141–144 region. It is puzzling why the loss of helicity did not start at the beginning of the helical region particularly taking the loss of this salt bridge into consideration. We speculate that the significant change in the interactions of the 133 position might have been translated into the 141–144 region through an allosteric path. In addition, since MeCP2 is an intrinsically disordered protein, it is reasonable to expect the DNA to induce different structural features for the WT and mutant MBD based on the sequence difference. Therefore, this reduced helical content might also be reflecting a different binding mode of the mutant to mDNA.

**Figure 4:**
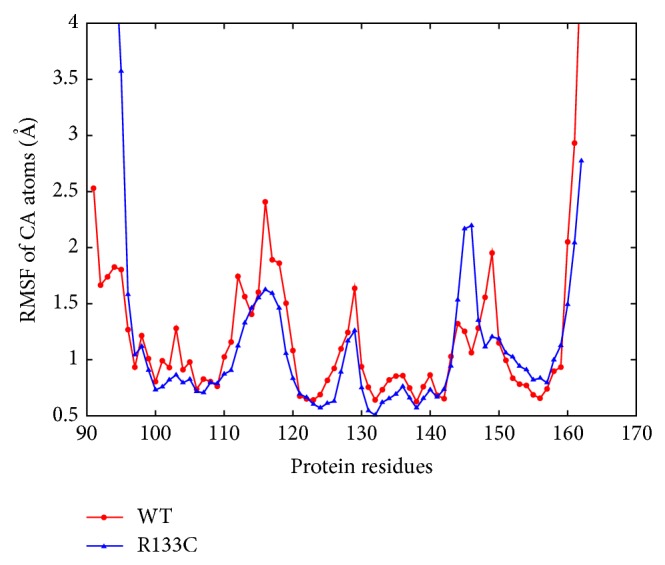
RMSF of CA atoms of all residues (91 to 162). Standard errors are omitted since they were negligibly small (on the order of 10^−3^).

Besides these structural changes, the dynamics of WT and R133C proteins were assessed through root mean square fluctuations (RMSF) of *C*_*α*_ atoms of each residue (Figure 4). The RMSF profiles can be considered as a measure of the average atomic mobility and on this plot, the peaks indicate areas of protein that fluctuate the most during the simulation. The protein was found to be rigidified upon the R133C mutation in all regions except the helix region. The most pronounced reduction in flexibility was seen in the two coil regions between the strands and the helix. Interestingly, the last two residues of the helical region and the next two residues showed a significant increase in flexibility. This is consistent with the structural changes in that region as shown in an RMSD plot in Figure 3 (top right panel) and also the secondary structure analysis in Figure 8. Therefore, the R133C mutation was found to have a profound effect on the structure and dynamics of the helical part of the protein at long time scales.

**Figure 5:**
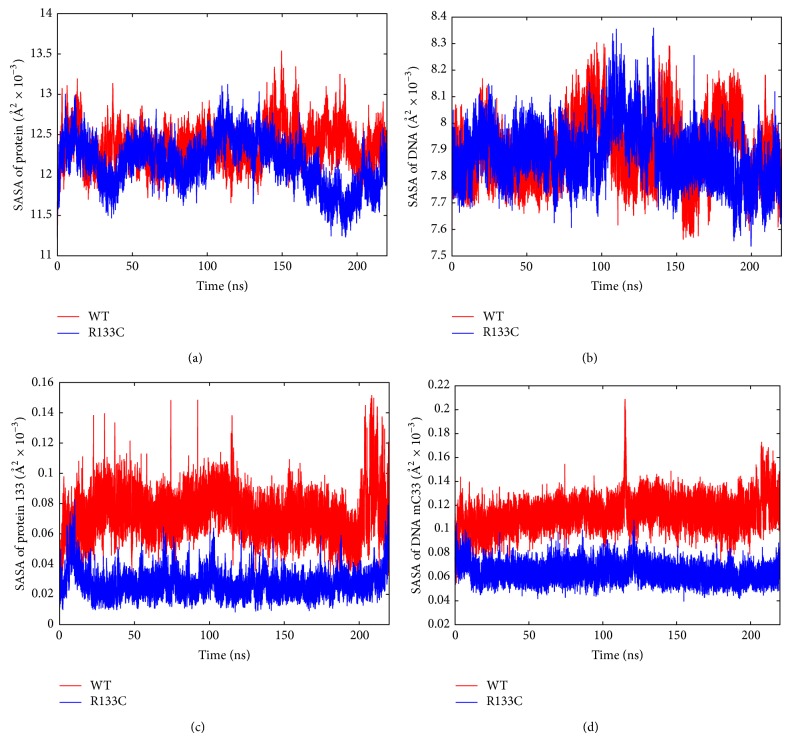
Time-dependent SASA profile of the full proteins (a) and residue 133 (c) and full DNA (b) and mC33 (d). WT is shown in red and R133C is shown in blue.

*(b) Impact of R133C Mutation on SASA and R*_*gyr*_. After examining the structural and dynamical properties of WT and R133C proteins, the possible consequences of the R133C mutation on hydration dynamics were monitored through solvent accessible surface area (SASA) profiles. Not surprisingly, the overall SASA profile for the proteins and the DNA have not been affected significantly by one residue change as shown in Figure 5 (top panels). However, the mutation caused a strong change in SASA of both the mutation position (133) and the methylated cytosine (mC33) of DNA Figure 5 (bottom panels). The visual inspection of the MD trajectories revealed that the coil region adapted a more buried conformation upon mutation. Interestingly, the R111 also became more buried as its SASA decreased by 23% upon mutation.

Following this, the compactness of the proteins was examined through computing their radius of gyration. Not surprisingly, the radius of gyration of the WT and R133C proteins did not show a significant change. Then, the two coil/turn regions in between strands and the helix were examined closely in regard to the radius of gyration profile. The 111–119 region did not show a significant change; however, as shown in Figure 5, the 126–134 region, in which the mutation took place, indicated an approximately 7% reduction in radius of gyration suggesting a more compact conformation for this coil region. This is consistent with the significant reduction of SASA in residue 133 upon mutation.

*(c) Impact of R133C Mutation on Interactions of MBD of MeCP2, DNA, and Water*. To quantify the significant SASA change of residue 133 further, the average number of hydrogen bonds that the proteins (WT and R133C) make with water was calculated. As shown in Table 2 (bottom table), the average number of hydrogen bonds in total decreased by 7% upon mutation. This overall decrease arose not only from the dramatic decrease (47%) of hydrogen bonds that the residue 133 makes with water, but also from other residues. For example, another key Arg at position 111 makes 17% less hydrogen bonds with water upon mutation of position 133. Also consistent with this is the 23% reduction in SASA of residue 111 as previously mentioned.

Turning now to protein-DNA interactions and how they are affected by R133C mutation, first, the hydrogen bonding network between the proteins and DNA was analyzed. The most obvious effect is that the direct hydrogen bond that Arg133 makes with the DNA is lost upon its mutation to Cys. Interestingly, the hydrogen bonding between R111 and DNA was also affected upon mutation indicating an 11% decrease. The degree of reduction in hydrogen bonding between the protein and DNA was found to be 21%. Also, the time-dependent contact area between the proteins (WT and R133C MBD) and DNA was monitored and an average overall decrease of 8% was observed in the R133C mutant system.

In addition, the time-dependent distance profile between residue 133 and mC33 of DNA indicated that the distance between the center of mass of residue 133 of protein and mC33 of DNA was lowered by about 11% (~1 Å). Therefore, geometrically, the Cys side chain is not sufficiently long to get as close to DNA compared to Arg and also considering the more buried and compact conformation that the 126–134 loop region adapts, a significant decrease in the interaction of protein and the DNA is expected upon R133C mutation.

**Figure 6:**
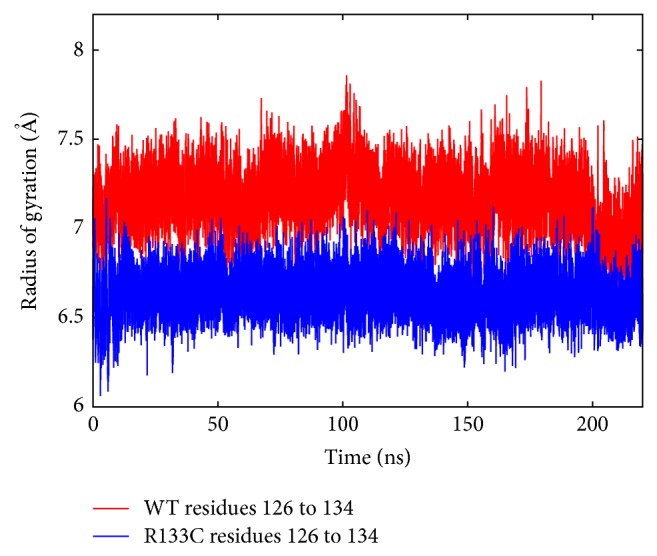
Time-dependent *R*_gyr_ profile of residues 126 to 134 for WT (red) and R133C (blue).

**Figure 7:**
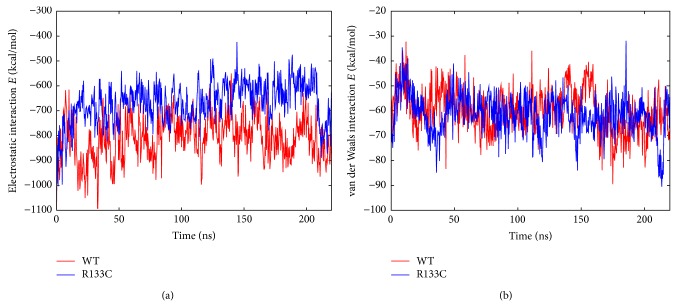
Electrostatic (a) and van der Waals (b) interaction energies between WT and DNA (red) and also between R133C and DNA (blue).

**Figure 8:**
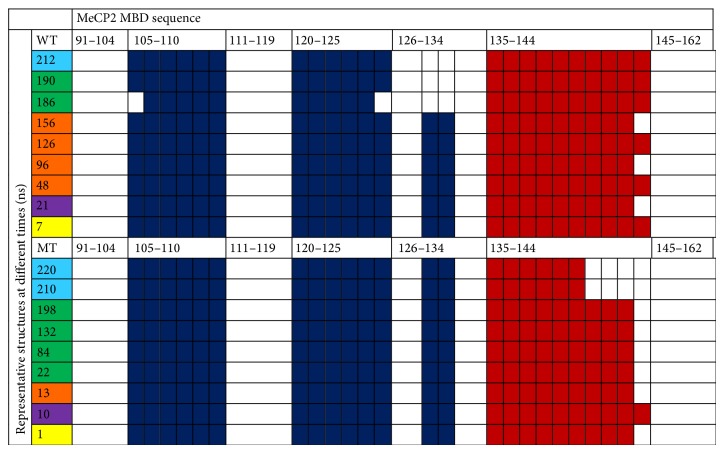
Secondary structure analysis for WT (top) and R133C (bottom). The left column shows representative structures at different stages (ns) of the simulation. The blue and red shaded areas represent strand and helix regions.

Finally, the interaction between the proteins and DNA, split into van der Waals and electrostatic interaction energy between DNA and proteins were calculated, as shown in Figure 7, to understand the manifestation of the loss of hydrogen bonding between the protein and DNA upon mutation. It should be clarified that the interaction energy is only a component of the total binding free energy and does not include desolvation penalty, for example. Consistent with the previous analyses, the R133C mutation was found to lower the interaction energy quite significantly. This is also consistent with previous EMSA (electrophoretic mobility shift assay) data suggesting a reduced binding of R133C to mDNA compared to WT [15]. The reduced interaction was found to be electrostatically driven as shown in Figure 6 and Table 3. The reduced interaction energy taken together with reduced hydrogen bonding between MBD and DNA might cause the reduced helicity in the R133C mutant. In other words, reduced interaction with the mDNA might cause reduced mDNA-induced stabilization and this may be manifested as the loss of helicity. Therefore, the R133C mutation was found to still maintain binding to DNA; however, its interaction is significantly reduced.

## 4. Conclusion

Comparative MD simulations of WT and mutant (R133C) MBD domain of MeCP2 were performed. The RTT mutation, R133C, was found to have pronounced effects on the structure and dynamics of MBD of the human MeCP2 protein and its interaction with DNA. The mutant was found to be stable and maintained binding to the methylated DNA, which is in agreement with experimental studies. However, the binding and the protein structure were significantly perturbed upon mutation. Two hydrogen bonds that residue 133 makes with mDNA were lost upon the R133C mutation, which may cause loss of specificity to mDNA in the mutant protein. Another important manifestation of the mutation is the reduced interaction energy between the mutant MBD and the DNA. Also, two salt bridges within the protein are lost upon the R133C mutation. The loss of salt bridges together with weakened interaction with DNA may cause a decrease in DNA-induced stability on MBD as the experiments suggest [15], which was found in our work to be manifested as the loss of helicity in the 141-142 region of mutant MBD. In addition, the protein hydration properties were significantly perturbed by R133C mutation. We speculate that the loss of helicity might have significant consequences in the interaction of MeCP2 with other protein partners subsequently adversely affecting its function, which may play a crucial role in RTT mechanism. Taken together, this suggests significant implications in understanding how the RTT-causing R133C mutation affects the protein structure at the molecular level. Further studies involving free energy calculations are underway to quantify the effects of this and other mutations on MBD of MeCP2-DNA interactions.

## Figures and Tables

**Table 1:** Salt bridges formed in the vicinity of R111 and R133 as monitored over the time-course of the simulations.

Salt bridges near R133 and R111		
	WT	R133C
E137-R133	✓	X
E137-K130	✓	✓
D121-R111	✓	✓
D121-K119	✓	X

**Table 2:** Average number of hydrogen bonds formed with DNA (top) and water (bottom) computed over the course of the trajectories. Standard errors are omitted since they were negligibly small.

	WT	R133C
Average number of hydrogen bonds with DNA		
Protein	7.21	5.70
Residue 133	0.63	0.0
Residue 111	0.74	0.66

Average number of hydrogen bonds with water		
Protein	81.8	76.2
Residue 133	1.68	0.79
Residue 111	0.47	0.39
DNA	150	156
mC33	2.70	2.73
mC8	2.69	2.59

**Table 3:** Average electrostatic and van der Waals interaction energies between protein and DNA.

Average interaction energy between protein & DNA (kcal/mol)		
	WT	R133C
Electrostatic	−840 ± 7	−711 ± 6
van der Waals	−56.4 ± 0.5	−59.2 ± 0.6
